# Low Serum Levels of Uric Acid are Associated With Development of Poststroke Depression

**DOI:** 10.1097/MD.0000000000001897

**Published:** 2015-11-13

**Authors:** Yingying Gu, Bin Han, Liping Wang, Yaling Chang, Lin Zhu, Wenwei Ren, Mengjiao Yan, Xiangyang Zhang, Jincai He

**Affiliations:** From the Department of Neurology, The First Affiliated Hospital of Wenzhou Medical University, Wenzhou, Zhejiang (YG, BH, LW, YC, LZ, WR, MY, JH), Beijing HuiLongGuan Hospital, Peking University, Beijing, P.R. China (XZ); and Menninger Department of Psychiatry and Behavioral Sciences, Baylor College of Medicine, Houston, TX (XZ).

## Abstract

Poststroke depression (PSD) is a frequent complication of stroke that has been associated with poorer outcome of stroke patients. This study sought to examine the possible association between serum uric acid levels and the development of PSD.

We recruited 196 patients with acute ischemic stroke and 100 healthy volunteers. Serum uric acid levels were tested by uricase-PAP method within 24 hr after admission. Neuropsychological evaluations were conducted at 3-month poststroke. The 17-item Hamilton Depression Scale was used to assess depressive symptoms. Diagnosis of PSD was made in accordance with DSM-IV criteria for depression. Multivariate analyses were conducted using logistic regression models.

Fifty-six patients (28.6%) were diagnosed as having PSD at 3 months. PSD patients showed significantly lower levels of uric acid at baseline as compared to non-PSD patients (237.02 ± 43.43 vs 309.10 ± 67.44 μmol/L, *t* = −8.86, *P* < 0.001). In multivariate analyses, uric acid levels (≤239.0 and ≥328.1 μmol/L) were independently associated with the development of PSD (OR, 7.76; 95% confidence interval [CI], 2.56–23.47, *P* < 0.001 and OR, 0.05; 95% CI, 0.01–0.43, *P* = 0.01, respectively) after adjustment for possible variables.

Serum uric acid levels at admission are found to be correlated with PSD and may predict its development at 3 months after stroke.

## INTRODUCTION

Poststroke depression (PSD) is a common and serious neuropsychiatric disorder following stroke. A recent pooled estimate indicates that PSD occurs in one-third of stroke survivors during follow-up.^1^ The presence of depression is associated with poorer functional outcomes,^2^ higher disability and mortality,^3^ reduced quality of life,^4^ and increased risk of recurrent stroke.^5^ Early recognition and diagnosis of PSD is of great importance in the reduction of stroke complications and better functional outcomes. However, the underlying pathophysiology of PSD remains unknown.

Oxidative stress characterized by as an imbalance between oxidants and antioxidants that results from overproduction of free radicals and reactive oxygen species, can affect various physiological functions, including the central nervous system. A fast growing body of evidence suggests the involvement of oxidative stress in the pathophysiology of depression.^6,7^ Uric acid is the ultimate catabolite of purine metabolism in the human body. On one hand, it functions as a pro-oxidant within the cells. Increased serum uric acid is a risk factor for cardiovascular and cerebrovascular diseases, particularly among patients with hypertension or diabetes mellitus.^8,9^ On the other hand, uric acid is one of the most important and robust antioxidants and it exerts neuroprotective effect via scavenging free radicals and reactive oxygen species.^10^ It has been reported that uric acid accounts for over 60% of the antioxidant capacity in the human body.^10^ Recent studies found low uric acid levels to be associated with a number of psychiatric disorders, including depression.^11,12^ The underlying pathophysiologic mechanism linking uric acid to depression is still unclear. Based on the involvement of oxidative stress in the pathophysiology of depression, 1 possible mechanism by which uric acid exerts its neuroprotective effect could be through a decrease in oxidative stress.^13^

Acute ischemic stroke leads to increased levels of oxidative stress and decreased levels of antioxidants, including uric acid.^14,15^ Notably, it is hypothesized that increased oxidative stress in cerebral tissues is implicated in the pathophysiology of PSD.^16^ Additionally, serum uric acid may be neuroprotective in acute ischemic stroke patients and may contribute to stroke outcome.^17,18^ Considering the involvement of uric acid in stroke as well as depression, we hypothesize that serum uric acid levels are related to the development of PSD.

## METHODS

### Study Population

Patients with acute ischemic stroke admitted to the First Affiliated Hospital of Wenzhou Medical University between October 2013 and September 2014 were consecutively screened and followed up for 3 months. Inclusion criteria included age between 18 and 80 years; acute stroke occurring within 7 days after stroke onset; magnetic resonance imaging or computerized tomography; the willingness and ability to give informed consent. Exclusion criteria were transient ischemic attack; subjects with a history of any central nervous system disease such as dementia or significant cognitive impairment, Parkinson's disease, tumor; subjects with a history of any psychiatric illness; subjects with severe aphasia, decreased level of consciousness, visual or auditory impairment; subjects with concomitant renal impairment. Meanwhile, 100 healthy volunteers without a history of psychiatric or renal impairment were recruited from a health survey. They were of similar gender and age distribution to the stroke patients. This study protocol was carried out in accordance with the Code of Ethics of the World Medical Association (Declaration of Helsinik) for experiments involving humans and approved by the Medical Ethics Committee of the First Affiliated Hospital of Wenzhou Medical University. Written informed consent was obtained from all patients.

### Clinical Variables

At admission, demographic data and history of conventional vascular risk factors were recorded. Stroke severity was assessed by trained neurologists using the National Institutes of Health Stroke Scale (NIHSS) within 24 hr of admission. Functional outcome was evaluated by the modified Rankin Scale (mRS) and the Barthel Index (BI) at discharge. Cranial computerized and magnetic resonance imaging were performed within 24 to 72 hr after admission in order to assess the site of the brain infarct. Blood sample was taken from all patients during 24 hr of admission. Serum uric acid was measured using uricase-PAP method in our hospital biochemistry department. The serum level ranging from 208 to 428 μmol/L in male patients and ranging from 155 to 357 μmol/L in female patients was categorized as normal. Serum uric acid was recorded and divided into 3 tertiles (≤239.0, 239.1–328.0, and ≥328.1 μmol/L), as the raw uric acid data were skewed.

### Assessment of PSD

Depression estimations were performed by a neurologist/psychiatrist who was blind to the laboratory results of stroke patients at 3 months after stroke. Stroke survivals should complete the 17-Hamilton Rating Scale for Depression (17-HAMD) at 3-month follow-up.^19^ Clinical depression was diagnosed on the basis of the Diagnostic and Statistical Manual of Mental Disorders, 4th edition (DSM-IV) criteria performed algorithms depended on psychiatric interview and neuropsychiatric examination.

### Statistical Analysis

The results were indicated as percentages for categorical variables, while continuous variables depending on their normal distribution were expressed as mean ± standard deviation (SD) or median (interquartile range, IQR). The Chi-squared test was employed for proportions and the normally distributed variables was compared using Student *t* test and analysis of variance (ANOVA), while the Mann–Whitney *U* test was employed for the asymmetrically distributed variables. Spearman's rank correlation was performed for bivariate correlation. The influence of serum uric acid levels on PSD was estimated by binary logistic regression analysis, after adjusting for potential confounding variables. Results were indicated as adjusted odds ratios (ORs) (95% confidence intervals, CIs). All statistical analysis was used SPSS for Windows, version 17.0 (SPSS Inc., Chicago, IL). Statistical significance was identified as *P* < 0.05.

## RESULTS

### Baseline Characteristics of Study Samples

The study cohort consisted of 251 individuals entry criteria. By the time of 3-month poststroke hospital follow-up, 2.0% (n = 5) had passed away and 19.9% (n = 50) declined due to missing data, leaving 196 patients. In this study population, 33.7% were female and the average age was 61.16 ± 10.10 years. Patients excluded from this study had a higher NIHSS score when compared with patients included in the study (*z* = −4.51, *P* < 0.001). There were no significant differences in age (*t* = −0.23, *P* = 0.82) and sex (χ^2^ = 0.08, *P* = 0.76).

### Main Findings

**FIGURE 1:**
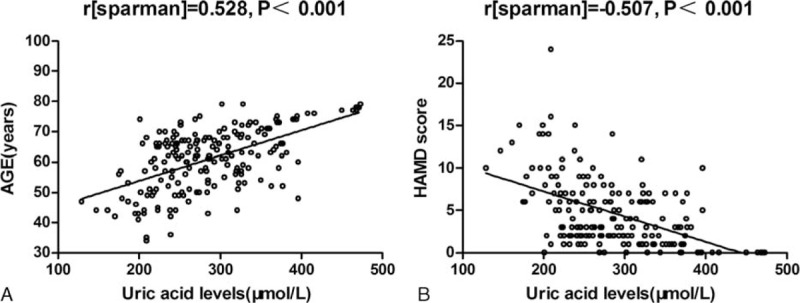
Correlation between serum uric acid levels and other predictors. (A) Correlation between the age of patients and serum uric acid levels; *r* [sparman] = 0.528, *P* < .001. (B) Correlation between the Hamilton Depression Scale (HAMD) score and serum uric acid levels; *r* [sparman] = −0.507, *P* < .001.

Of the 196 subjects who formed the study sample, 56 (28.6%, 24 men, 32 women) were diagnosed with PSD. The average hospitalization mean (±SD) time per patient was 10.40 ± 3.25 days. The mean (±SD) uric acid levels at baseline were significantly lower in stroke patients as compared to normal controls (288.51 ± 69.57 vs 344.46 ± 88.06 μmol/L, *t* = 5.53, *P* < 0.001). There was a positive correlation between serum levels of uric acid and age of the patients (*r* = 0.53, *P* < 0.001; Fig. 1A). There was a negative correlation between serum levels of uric acid and HAMD scores (*r* = −0.51, *P* < 0.001; Fig. 1B). There was a significant difference in serum uric acid levels between men and women (*t* = −4.60, *P* < 0.001). The PSD patients were younger (*z* = −2.56, *P* = 0.01) and had a more severe stroke (*z* = −3.82, *P* < 0.001) and poorer functional outcomes (*z* = −6.60, *P* < 0.001). No association was found between lesion location and PSD (Table 1).

**TABLE 1:**
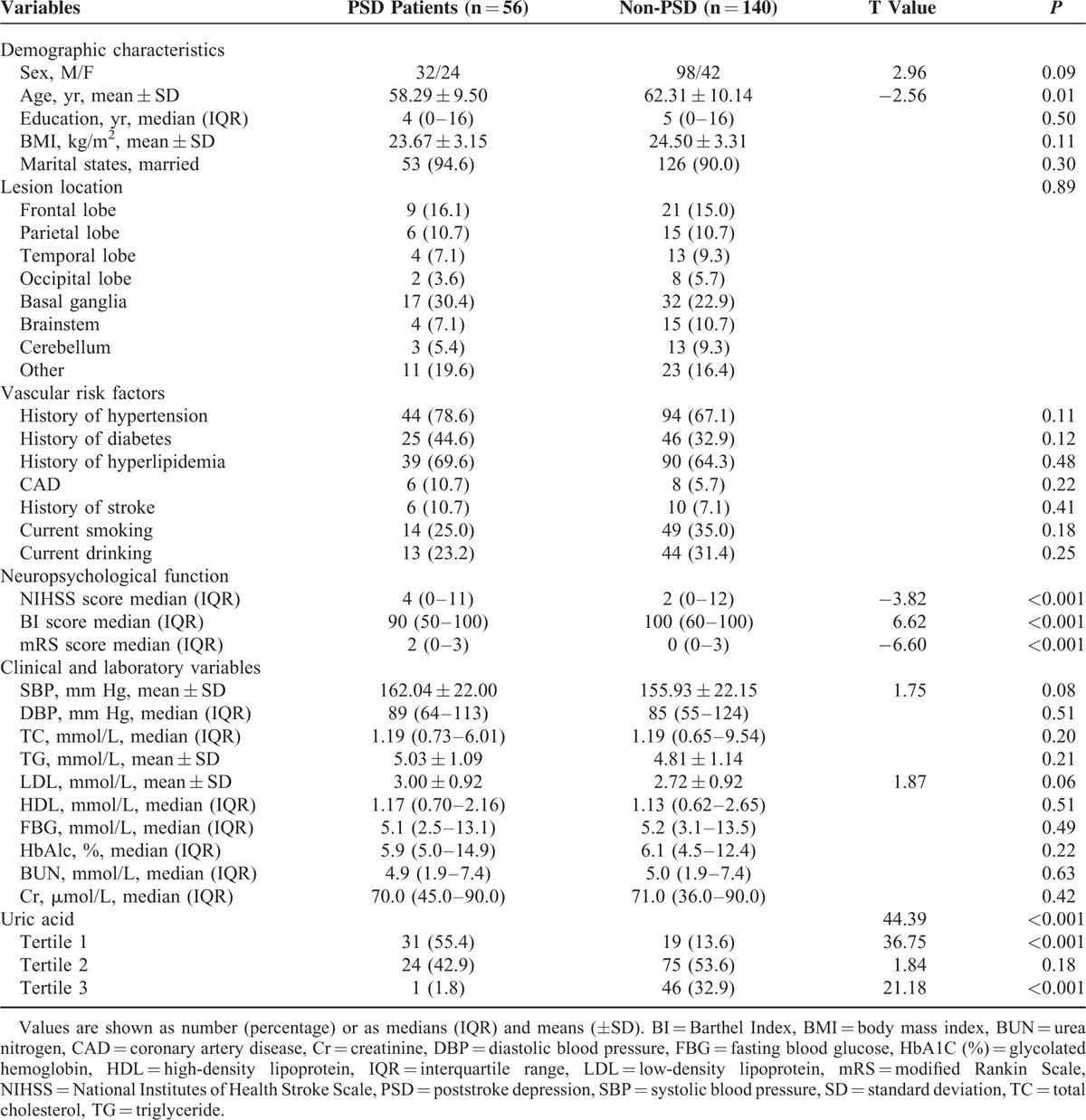
Clinical and Demographic Characteristics of the Samples Under Study

Serum median uric acid levels were significantly decreased at admission in PSD patients than non-PSD patients (237.02 ± 43.43 vs 309.10 ± 67.44 μmol/L, *t* = −8.86, *P* < 0.001). Additionally, significant differences were observed between the PSD patients and stroke patients without depression in uric acid level tertiles (χ^2^ = 44.39, *P* < 0.001). Indeed, the proportion of serum uric acid in the lowest tertile (≤239.0 μmol/L) was significantly elevated in the PSD patients (χ^2^ = 36.75, *P* < 0.001), meanwhile the proportion of serum uric acid in the highest tertile (≥328.1 μmol/L) was significantly decreased in the PSD patients (χ^2^ = 21.18, *P* < 0.001; Table 1). With all stroke subjects taken as a whole, stroke patients with depression presence as a dependent variable, factors with *P* < 0.10 in the univariate analysis as independent variables in the logistic analysis. Results found that uric acid levels (≤239.0 and ≥328.1 μmol/L) were independently associated with the presence of PSD (OR, 7.76; 95% CI, 2.56–23.47, *P* < 0.001 and OR, 0.05; 95% CI, 0.01–0.43, *P* = 0.01, respectively). Moreover, the mRS scores at discharge were significantly associated with the presence of depression 3 months after stroke (OR, 2.49; 95% CI, 1.20–5.16, *P* = 0.01; Table 2).

**TABLE 2:**
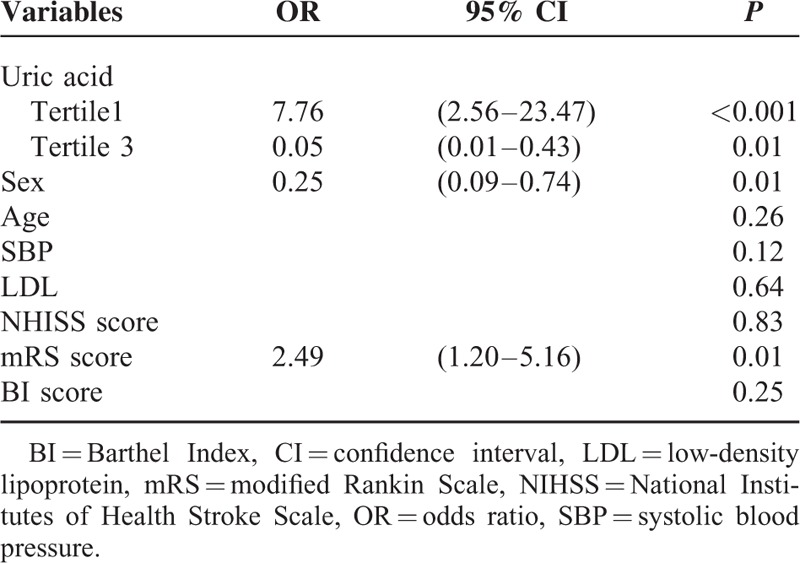
Adjusted OR of Depression for Serum Uric Acid Levels at Admission in the Stroke Patients

## DISCUSSION

To our knowledge, this is by far the first study explored the possible relationship between serum uric acid levels at admission and the presence of depression 3 months after stroke. Our results indicated that uric acid is a powerful biological marker of presence of PSD at 3 months after stroke. Therefore, it may be used as a useful novel therapeutic target for the treatment of PSD.

In the present study, we found that 28.6% of stroke patients were diagnosed as having major depression at 3 months after stroke, which is consistent with the results of recent researches.^1,5^ Currently, despite the already vast literature, it remains difficult to determine the true prevalence of PSD, possibly due to the different study designs, methods used to diagnose PSD, time of evaluation, and source of patient recruitment, together with the racial disparities in PSD detection.^4^

Serum uric acid levels are affected by both age and gender. In the present study, we found that men had higher serum uric acid levels when compared to women. There was a significant positive correlation between age of stroke patients and serum uric acid levels. These results broadly agree with previous studies.^20,21^ Furthermore, serum levels of uric acid at admission were found to be significantly lower in stroke patients than in normal subjects, in agreement with previous reports.^22,23^

Importantly, we found that serum uric acid levels at admission were independently associated with the development of PSD at 3 months after stroke. As mentioned earlier, a fast growing body of studies have reported low uric acid levels in patients with psychiatric disorders, including depression.^11,12^ They also found that serum levels of uric acid in patients with depression normalized after treatment with antidepressants. In addition, a significant inverse relationship between serum uric acid levels and Zung Self-Rating Depression Scale scores was observed in a population-based study.^24^ Recently, an important association between low serum uric acid levels at baseline and depression/anxiety was found in patients with Parkinson's disease, which may suggest a role for uric acid in both presence and progression of mood disorders in Parkinson's disease.^25^ In this follow-up study, we also found high uric acid levels at baseline were protective against developing PSD at 3 months after stroke. A large number of studies in stroke patients have suggested that elevated uric acid levels were associated with good outcomes.^17^ Recently, a phase 2 clinical trial with inosine in Parkinson's disease demonstrated a potential efficacy of increased uric acid on mood disorders as evaluated on the Geriatric Depression Scale.^26^

The exact role of uric acid in the pathophysiology of depression remains unknown. Recently, oxidative stress has gained attention as one of the potential mechanisms in the pathophysiology of depression. Oxidative stress causes damage to lipids, proteins, DNA, and mitochondria and eventually causes neurotoxicity and neurodegeneration in major depression.^27–30^ Moreover, oxidative stress is closely associated with the inflammatory response. Expression of pro-inflammatory cytokines is increased in reaction to oxidative stress and oxidative stress in turn enhances the inflammatory response. Inflammatory response also plays an important role in the pathogenesis of depression.^31^ Uric acid has multiple antioxidant roles that include the scavenging of free radicals and reactive oxygen species, in addition to the chelation of transition metals, the suppression of the Fenton reaction, and the prevention of lipid peroxidation.^32,33^ Furthermore, uric acid is capable of suppressing inflammatory cascade, decreasing blood–brain barrier permeability, and diminishing central nervous tissue damage and neuronal death.^34,35^ Therefore, one of the mechanisms linking uric acid to depression is the neuroprotective effect of uric acid via providing a defense against oxidative stress.^13^

Acute ischemic stroke triggers overproduction of free radicals and reactive oxygen species in ischemic brain tissue and facilitates lipid peroxidation, mitochondrial dysfunction, excitotoxicity, and inflammatory response.^36^ As a result, pro-inflammatory cytokines, matrix metalloproteinases, and endothelial adhesion molecules are produced, which may further amplify oxidative stress and brain tissue damage. In acute stage of stroke, uric acid may exert antioxidant protection against free radical damage. A large number of studies have shown a negative correlation between serum uric acid levels and stroke outcomes.^17,37^ A recent animal study demonstrated that uric acid administered after thromboembolic stroke is neuroprotective in the rat brain and it strengthens the benefits of recombinant tissue plasminogen activator.^38^ More recently, a randomized clinical trial showed that uric acid therapy was related to a decrease in glucose-driven oxidative stress and improved outcome in patients with hyperglycemia in acute stage.^39^ Therefore, given its involvement in stroke as well as depression, uric acid may play an important role in the development of PSD.

We found that physical disability measured by mRS at discharge was a risk factor of the development of PSD, which broadly agrees with the findings of previous studies.^4,40^ Our results also demonstrated that female gender was independently associated with PSD. A pooled estimate also indicates that PSD is slightly more common among women than men.^41^ We did not detect any significant association between the development of PSD and other potential variables, including lesion location and NHISS scores, although previous studies have provided conflicting findings on these aspects.

There are several limitations in the present study. First, the fairly rigid inclusion criteria resulted in a relatively small sample size and, in turn, reduced the statistical power of the study. Second, both patients with severe aphasia and patients who died before the 3-month follow-up were not included, which might make us underestimate the actual prevalence of PSD. Third, serum levels of uric acid were measured only 1 time at admission, further studies are needed to evaluate how uric acid levels change across time following stroke and whether its levels increased at later points improve stroke outcomes. Lastly, the study subjects were not randomly selected and came from only 1 clinic. Therefore, our results may not be readily generalized to other Chinese patients. Further research is needed.

In summary, in spite of these limitations, our study demonstrates an important association between serum uric acid levels at admission and the development of PSD at 3 months after stroke. Further studies on larger populations should be encouraged to confirm this association, which may provide novel proposal for the prevention of PSD, the treatment of PSD, or both.
